# Long-Bone-Regeneration Process in a Sheep Animal Model, Using Hydroxyapatite Ceramics Prepared by Tape-Casting Method

**DOI:** 10.3390/bioengineering10030291

**Published:** 2023-02-24

**Authors:** Lenka Kresakova, Lubomir Medvecky, Katarina Vdoviakova, Maros Varga, Ján Danko, Roman Totkovic, Tatiana Spakovska, Marko Vrzgula, Maria Giretova, Jaroslav Briancin, Veronika Šimaiová, Marian Kadasi

**Affiliations:** 1 Department of Morphological Disciplines, University of Veterinary Medicine and Pharmacy in Kosice, Komenskeho 73, 041 81 Kosice, Slovakia; 2 Division of Functional and Hybrid Systems, Institute of Materials Research of SAS, Watsonova 47, 040 01 Kosice, Slovakia; 3 Hospital AGEL Kosice-Saca, Lucna 57, 040 15 Kosice, Slovakia; 4 Department of Anatomy, Faculty of Medicine, Pavol Jozef Safarik University in Kosice, Trieda SNP 1, 040 11 Kosice, Slovakia; 5 Institute of Geotechnics SAS, Watsonova 45, 040 01 Kosice, Slovakia; 6 Clinic of Ruminants, University of Veterinary Medicine and Pharmacy in Kosice, Komenskeho 73, 041 81 Kosice, Slovakia

**Keywords:** hydroxyapatite, ceramic, tape-casting, sheep, biocompatibility, biodegradation

## Abstract

This study was designed to investigate the effects of hydroxyapatite (HA) ceramic implants (HA cylinders, perforated HA plates, and nonperforated HA plates) on the healing of bone defects, addressing biocompatibility, biodegradability, osteoconductivity, osteoinductivity, and osteointegration with the surrounding bone tissue. The HA ceramic implants were prepared using the tape-casting method, which allows for shape variation in samples after packing HA paste into 3D-printed plastic forms. In vitro, the distribution and morphology of the MC3T3E1 cells grown on the test discs for 2 and 9 days were visualised with a fluorescent live/dead staining assay. The growth of the cell population was clearly visible on the entire ceramic surfaces and very good osteoblastic cell adhesion and proliferation was observed, with no dead cells detected. A sheep animal model was used to perform in vivo experiments with bone defects created on the metatarsal bones, where histological and immunohistochemical tissue analysis as well as X-ray and CT images were applied. After 6 months, all implants showed excellent biocompatibility with the surrounding bone tissue with no observed signs of inflammatory reaction. The histomorphological findings revealed bone growth immediately over and around the implants, indicating the excellent osteoconductivity of the HA ceramic implants. A number of islands of bone tissue were observed towards the centres of the HA cylinders. The highest degree of biodegradation, bioresorption, and new bone formation was observed in the group in which perforated HA plates were applied. The results of this study suggest that HA cylinders and HA plates may provide a promising material for the functional long-bone-defect reconstruction and further research.

## 1. Introduction

During the last decades, many researchers have investigated the biological process of bone regeneration in order to develop better strategies for bone healing [1,2]. The standard therapy for patients suffering from severe long-term problems, incomplete healing, nonunion healing (5–10% of fractures), or major bone defects is bone grafting using an autograft or allograft. Autologous grafts are collected mainly from other areas in the body (taken from the iliac crest, proximal tibia, ribs, etc.) and then transplanted into large fractures. There are many problems and obstacles associated with these procedures, e.g., treatment is limited by bone volume, recurrent fractures, soreness, associated complications such as infection, local haematoma, poor integration, donor site shortage, prolonged surgery time, donor site morbidity, and vascular problems [3,4,5]. Generally, allografts have low osteogenicity, low mechanical resistance, can evoke immunologic reactions, and pose a risk of graft rejection [6,7]. Therefore, a more sustainable and long-term treatment plan is necessary. For this purpose, bone graft replacements have been created to help treat damaged bones. The main strategies for bone repair are synthetic prostheses, carriers combined with various active molecules, cell therapy, biomimetic prostheses, bioactive polymers, inorganic composites, and others [8]. Biomaterials used in bone research should meet the main conditions of biocompatibility, biological stability, and the absence of immune response [9]. Despite a long period of research efforts, an ideal grafting material does not exist [10].

Calcium-phosphate (CaP)-based biomaterials have a composition comparable to bone minerals; therefore, they are able to induce biological responses similar to those found in bone healing [11]. CaP-based bone substitutes, especially tricalcium phosphate (αTCP and βTCP), hydroxyapatite (HA), and biphasic CaP-based bioceramics, present an appropriate choice in the field of bone tissue engineering and appear to be a suitable alternative to bone grafts in surgical bone replacement [12]. The composition of CaP is closely related to bone constitution; for that reason, CaP bone substitutes are successfully used in different clinical applications, e.g., artificial bone grafts, bone augmentations, maxillofacial reconstruction, bone replacements after tumour surgery, and orthopaedic and maxillofacial surgery [13,14,15]. CaP-based bioceramics are considered bioactive, biodegradable, biocompatible, and osteoconductive [5,16]. Certainly, it is necessary to mention that not all types of CaP have the same attributes and abilities. Some of them are able to degrade in short time in vivo, while others are stable. Although it is generally accepted that CaPs have osteoconductive properties [17,18], a number of studies have reported the osteoinductive capabilities of some types of CaP-based bioceramics [19,20,21,22,23,24,25,26,27]. The main advantages of CaP-based bioceramics are the chemical resistance, nonreactivity, nontoxicity, and degradability through cellular, biological, and chemical processes [14]. CaPs offer appropriate surface properties (e.g., roughness, porosity, and solubility), allowing the adhesion and proliferation of osteoblasts at the implant site and the increase in osteogenesis [5].

HA is one of the naturally occurring types of CaP that represents the essential ingredient and the largest amount of inorganic constituent in bones [28] and, related to that, provides the rigidity of the bone [29,30]. HA was reported as nontoxic, only minimally inflammatory, and osteoconductive [31,32]. Many previous findings have shown that HA has a very good biocompatibility, biomimetic character [6,33,34], and the functional characteristics that facilitate cell growth and the formation of new bone. Among the various synthetic biomaterials, HA gives the best results [35]. This material can be used alone or in combination with other biomaterials for bone reconstruction in many clinical applications [36,37]. HA can be synthesised by various methods and can be easily prepared in a laboratory [38]. For these properties, HA is still an interesting biomaterial that is used in bone tissue regeneration. The design of bioceramics also represents a new direction for bioceramics science [39].

As a result, and due to the positive scientific evidence, the main objective of this study was to observe the effect of the originally prepared HA ceramics (HA-perforated plates, HA-nonperforated plates, HA cylinders) on bone tissue regeneration. The HA ceramics were prepared using a tape-casting method which allows variation in the shape of samples after packing HA paste to the 3D-printed plastic form. The use of HA in cranial applications is relatively common and little or no stress and pressure develops under these conditions. In our study, we used a sheep long-bone-defect model. Our preclinical investigations focused on the effect of HA ceramics on the healing of bone defects, biocompatibility, biodegradability, bioresorption, osteoconductivity, osteoinductivity, and bonding to the surrounding tissue. After the in vivo application of HA cylinders and HA plates, we expected the formation of new bone tissue with properties similar to the original bone.

## 2. Materials and Methods

### 2.1. HA Synthesis and Preparation of HA Ceramics

Hydroxyapatite powder was synthesised by the precipitation of 0.5 M Ca(NO_3_)_2_·4H_2_O (Sigma-Aldrich, St. Louis, MO, USA, analytical grade) solution and 0.5 M (NH_4_)_2_HPO_4_ (Sigma-Aldrich, analytical grade) solution with a molar ratio of Ca/P equal to 1.66. The aqueous solution of Ca^2+^ ions was applied slowly dropwise into the phosphate aqueous solution for 1.5 h using a peristaltic pump at pH close to 10.5, achieved by adding NH_3_(aq) (1:1) at 25 °C. Rotation speed of the magnetic stirrer was 450 rpm. The precipitates were allowed to age for 72 h at room temperature, with 24 h exchange of the solution above the HA layer. Then, the HA precipitates were washed with distilled water, ethanol, and filtered. The obtained hydroxyapatite powders (HAPs) were dried at 110 °C for 4 h, crushed, and sieved (Mesh 250). HA ceramics were prepared by a tape-casting method. First, the synthesised HAP was mixed with hydrogel (2 wt% carboxymethyl cellulose + 1 wt% polyacrylic acid) + glycerol for 5 min in a planetary ball mill (Fritsch, agate balls and vessel). The ratios of hydrogel to glycerol and HAP/hydrogel were 6:1 and 1:1, respectively. The obtained paste was moulded to polylactic acid forms printed on 3D printer (3D printer da Vinci 3 in 1, XYZ printing Inc, Thailand), dried at 37 °C for 12 h, removed from the form, and dried at 100 °C for 1 h and 120 °C for another hour. The final green samples were sintered at 1250 °C for 2 h at a heating rate of 5 °C/min.

### 2.2. Characterization of HA Ceramics

The green samples had plate (25 × 10 × 3 mm), cubic (10 × 10 × 10 mm), or cylindrical (8 mm in diameter and 15 mm in length) shapes. The apparent density of the sintered ceramic samples was determined by calculation from the measurement of dimensions and mass of cubic-shaped samples. The theoretical density of the HA was 3.15 g·cm^−3^. The phase composition of the samples was analysed by X-ray diffraction (Philips X_PertPro). The microstructure of the scaffolds was observed using a scanning electron microscopy (SEM, JEOL FE SEM JSM-7000F). The compressive strength of the ceramic samples was measured by a LR5K Plus (Lloyd Instruments, Ltd., Fareham, UK) at the loading rate of 1 mm.min^−1^. The ceramic samples were then sterilised in a thermostat at 160 °C/1 h.

### 2.3. In Vitro Testing

The substrates in the form of pellets (~6 mm in diameter and 0.5 mm in height) were soaked in PBS solution for 5 min at 37 °C, placed to the wells of a 96-well suspension plate, and 200 μL of the complete culture α-modification minimum essential Eagle medium (αMEM) (10% foetal bovine serum, 1% ATB-ATM and osteogenic supplements: β-glycerophosphate 10 mM; ascorbic acid 50 μmL^−1^ and dexamethasone 50 nM, obtained from Sigma) containing 1.0 × 10^4^ preosteoblastic murine MC3T3E1 cells (ATCC CRL-2593, Manassas, VA, USA) were carefully seeded on each disc. The density, distribution, and morphology of the MC3T3E1 cells grown on the tested discs for 2 and 9 days were visualised with fluorescent live/dead staining (fluorescein diacetate/propidium iodide) under an inverted optical fluorescence microscope (Leica DM IL LED, blue filter).

### 2.4. Animals, Surgical Procedures, and Postsurgical Management

**Figure 1:**
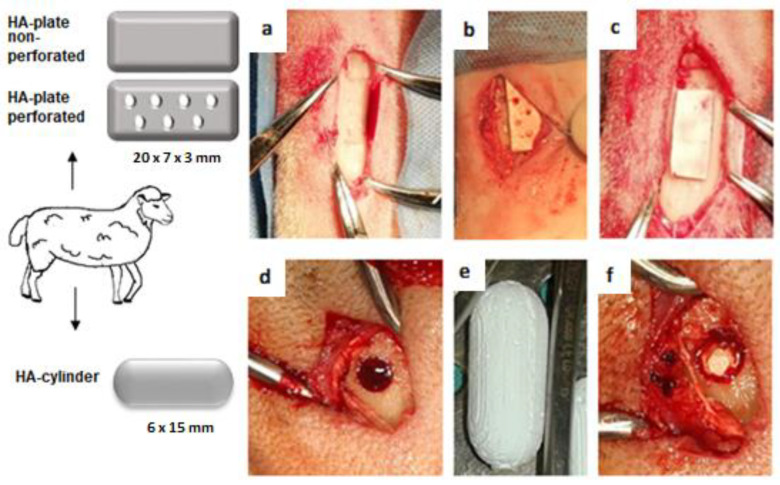
Surgical procedure. (**a**,**d**) Bone defect in metatarsal bone. (**b**) Perforated HA plate inserted to the bone defect. (**c**) Nonperforated HA plate inserted to the bone defect. (**e**) HA cylinder. (**f**) HA cylinder inserted to the bone defect.

Twelve adult healthy female sheep of the Valachian/Merino breed were used in this study. Ethical approval was obtained from the State Veterinary and Food Administration of the Slovak Republic no. 2220/17-221.The average weight of animals was 65.7 kg (range: 59–73 kg), and at the time of surgery the sheep were from 2 to 2.5 years old. General anaesthesia was induced by an intramuscular injection of butorphanol (0.1 mg/kg, Butomidor 10 mg/mL, Richter Pharma, Wels, Austria), medetomidine 0.02 mg/kg (Cepetor 1 mg/mL, CP-Pharma Handelsgesellschaft, GmbH, Burgdorf, Germany), and ketamine 8 mg/kg (Ketamidor 100 mg/mL, Richter Pharma, Wels, Austria) administered intravenously. In all sheep, the area around an impending operation on the left hind limb was shaved and prepared with a Betadine and alcohol solution using a sterile technique. We made a 3–5 cm long skin and soft tissue incision in the area of the planned defect. All animals were divided into 2 groups. Group 1 (n = 6): bone defects with a diameter of 6 mm and a depth of 15 mm were created by drilling in the distal end of the metatarsal bone, one defect in each animal. After that, an HA cylinder (15 × 6 mm) was implanted in the bone defect from the median side. Group 2 (n = 6): bone defects with dimensions 20 mm (length), 7 mm (width), and 3 mm (depth) were created by surgical chisels and filled with an HA plate (20 × 7 × 3 mm). Three HA plates were perforated by 7 holes with a diameter of 1 mm, while the remaining 3 were nonperforated (Figure 1). The original bone was partially removed, leaving an incomplete gap between the bones. All biomaterials completely filled the defect. After implanting the HA cylinders and HA plates, the surrounding tissues and skin were sutured. The surgical wound was covered by an aluminium fluid spray. The lack of bone fractures and correct performance of the surgical procedure was validated by X-ray (Philips Digital Diagnost, Delft, The Netherlands).

After the surgical operation, the sheep were returned to cages and allowed to move freely without external support and fixation. Every second day, oxytetracycline dihydricum 20 mg/kg (Alamycin LA a.u.v., Norbrook, Newry, UK) was administered for 7 days after the surgery intramuscularly. Flunixin meglumine 2.2 mg/kg (Flunixin a.u.v., Norbrook, Newry, UK) was administered for postsurgical pain management intramuscularly, once a day for 7 days, and then as needed. All animals were euthanised 6 months after the surgery, according to a standard protocol, with xylazine 0.2 mg/kg (Rometar 20 mg/mL inj., Bioveta, Nitra, Slovak Republic) administered intramuscularly and ketamin 2 mg/kg (Narkamon 100 mg/mL inj., Bioveta) administered intravenously. The bones were examined by X-ray and CT and consequently evaluated histomorphologically and immunohistochemically. The results were compared with a control group consisting of physiological bone tissue.

### 2.5. Histological and Immunohistochemical Processing

Bone tissue samples for histological examination were fixed in neutral formalin for 1–2 weeks. After washing in running water, the samples were placed in a chelaton solution for 4 weeks in a thermostat at 56 °C. The chelaton solution was changed once a week. At the end of the descaling process, the samples were embedded in paraffin. Sections were cut at 7 μm using a microtome (Leica, Bensheim, Germany) and stained by haematoxylin–eosin and Masson’s trichrome. Haematoxilin–eosin staining was used to assess the cell morphology and structure of the new bone. To examine the maturity of the newly formed bone, Masson’s trichrome staining was performed. Histologic sections were evaluated by a light microscope Olympus CX 43 (Olympus Corporation, Tokyo, Japan) and 300 MIPromicam digital camera (Promicra, Prague, Czech Republic). Evaluation was performed by two observers on five samples per each bone defect.

The expression of type I collagen (COL1) was investigated by immunohistochemical staining. Bone specimens were decalcified in chelaton during a 3-week period and the specimens were then washed in sterile distilled water for several hours. After washing, the bone tissue was dehydrated in alcohol and embedded in paraplast. Subsequently, two slices with 7 μm thickness were taken from the implantation site for histological and immunohistochemical analysis. An immunohistochemical reaction was performed to demonstrate the presence of COL1 using a primary Rabbit polyclonal anticollagen I antibody (Abcam, ab233080) and a secondary DB DET SYS kit. The DB detection kit was a rabbit/mouse dual system (Biotech). DAB (3,3′-diaminobenzidine) (DAKO) was used to visualize the reaction. Finally, the cell nuclei were stained with acidic Mayer’s haematoxylin.

### 2.6. X-ray and CT Analysis

X-ray analysis was done employing X-ray equipment (Philips Digital Diagnost, The Netherlands) at the exposure settings of 55–60 kV and 1.8–4.9 mAs and the pixel size of 0.133 mm. CT scans were obtained using a CT (Philips Brilliance 40-slice CT, Delft, The Netherlands) with the following scanning parameters: 120 kV, 250 mA, 300 mAs, pixel size 0.283 mm, thickness of slices 2 mm. We evaluated the presence of nonresorbed and nondegraded biomaterial, pathological and inflammatory changes, and the density of the newly formed bone tissue compared to the surrounding physiological bone. The bone density was evaluated using Hounsfield units (HU).

### 2.7. Ca/P Ratio

The Ca/P ratio in newly formed bones and native bone was determined after dehydration in ethanol and coating with carbon by a field emission scanning electron microscopy (SEM) (JEOL FE SEM JSM-7000F, Jeol Ltd., Tokyo, Japan) equipped with the energy-dispersive X-ray analyser (EDX) (INCA, Oxford Instruments, Abingdon, UK).

### 2.8. Statistical Analysis

Statistical analysis for differences between groups was performed using an unpaired *t*-test (GraphPad Prism 7.0 for Windows, GraphPad Software, San Diego, CA, USA). All data were expressed as means and standard deviations of means (SD). Differences were considered statistically significant at the levels of * = *p* < 0.05; ** = *p* < 0.01; *** = *p* < 0.001.

## 3. Results

### 3.1. Microstructure, Properties, and Live/Dead Staining of Ceramic Samples

**Figure 2:**
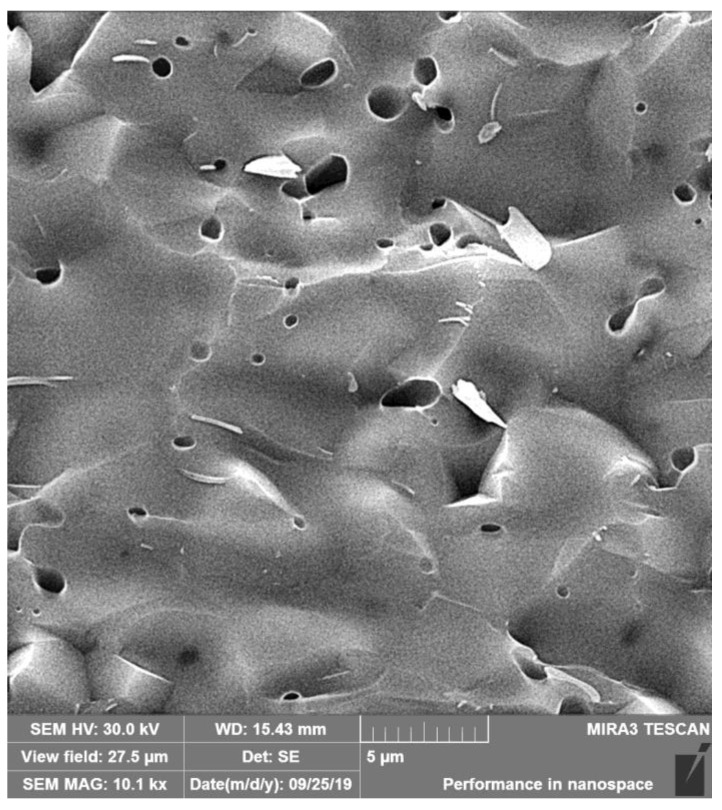
Microstructure of the fractured ceramic sample. A low fraction of 0.5–2.5 µm spherical pores is visible in the microstructure.

**Figure 3:**
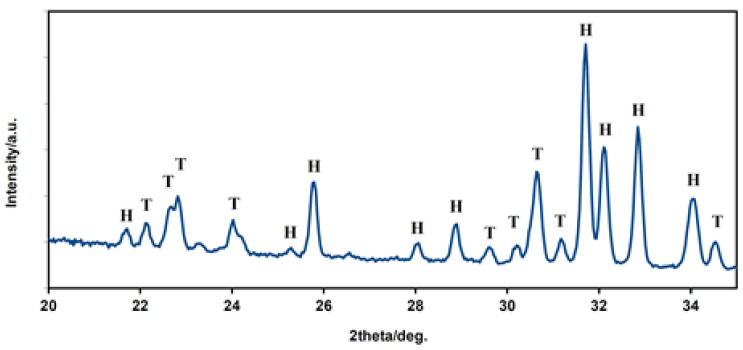
XRD patterns of HAP biphasic ceramics. The XRD phase analysis identified biphasic CaP ceramics with HA (H) as the main phase and α-TCP (T) as the secondary phase. The composition corresponds to a small chemical nonstoichiometry of HAP powder during synthesis. H—hydroxyapatite, T—α tricalcium phosphate.

**Figure 4:**
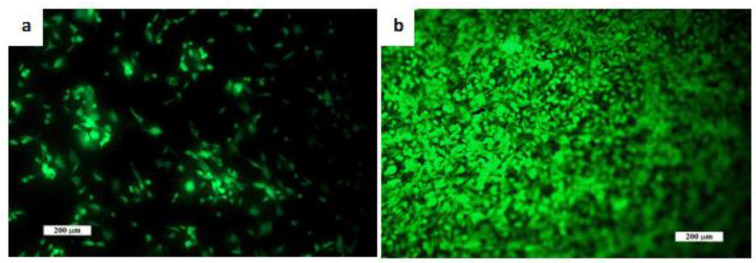
Distribution and morphology of an osteoblast cultured on ceramic samples for (**a**) 2 days and (**b**) 9 days (live/dead staining). The growth of the cell population is clearly visible and (**b**) a dense layer of osteoblasts covers the entire ceramic surface. Cell adhesion and proliferation was very good, and no dead cells were observed. Scale bar: 200 µm.

The dense microstructure of the ceramic sample is shown in Figure 2. In the microstructure, a low fraction of 0.5–2.5 µm spherical pores is visible in the image, located mainly at the boundaries between three adjacent grains and formed by coalescence during sintering. The grain boundaries are relatively difficult to distinguish on fracture surfaces in a transgranular fracture mode, but from close observation, we believe that the HAP grain size did not exceed 5 µm. However, the sintering process was stopped before the final phase of densification of ceramics, when individual micropores were gradually eliminated from the microstructure. The relative density of the HA ceramic samples reached 84 ± 3% of the theoretical HAP density. The XRD phase analysis (Figure 3) identified biphasic CaP ceramics with HA as the main phase (JCPDS 72-1243) and α-tricalcium phosphate (αTCP) as the secondary phase (JCPDS 29-0359). This composition would correspond to a small chemical nonstoichiometry of HAP powder during synthesis (e.g., as a result of substitution of carbonates for hydroxyl or phosphate group) with formation of calcium-deficient HAP. The average compressive strength of the samples was 71 ± 5 MPa, which is much higher than that of a cancellous bone (up to 15 MPa) but lower compared to a compact bone (up to 200 MPa) [40]. Nevertheless, it is a value sufficient for utilization of the ceramics as the bone defect filling material. Figure 4 shows a good adherence and spreading of osteoblastic cells on the ceramic surfaces and the absence of dead cells after culturing for 2 and 9 days. The growth of the cell population is clearly visible from the comparison of the 2- and 9-day cultured samples as a dense layer of osteoblasts covering the entire ceramic surface. These facts demonstrated the noncytotoxic character of the ceramic surfaces and the appropriate texture as well as the physicochemical properties of the samples. Obviously, αTCP is more soluble than HA and can significantly stimulate the osteogenic activity of osteoblasts in contact with the biphasic ceramic system [41].

### 3.2. General Behaviour of Animals

All sheep recovered well after anaesthesia and surgery with no serious complications. The general behaviour and condition were not negatively affected by the surgical intervention, and no abnormalities were observed regarding water and food intake behaviour. Visual examination showed a slight degree of lameness during the first 3 days after surgery with gradual improvement up to 10 days. Vital signs (body temperature, pulse rate, respiration rate) corresponded to the normal values. All animals survived without clinical signs of visually detectable pathological changes (damage) of wound. There were no macroscopic signs of infection, and no inflammatory processes were detected in any of the monitored sheep. All implants were well positioned at the surgery sites without any dislocation or loosening. No apparent breakage or other damage to the implants was observed. All HA cylinders and HA plates were well tolerated in vivo throughout the 6-month duration of the experiment.

### 3.3. Histomorphological and Imunohistochemical Analysis

#### 3.3.1. HA Cylinders

All implanted HA cylinders showed excellent biocompatibility with the surrounding bone tissue. We did not detect a fibrous interface, fibrous capsule, or pathological changes at the interface of the biomaterial and the bone. No cellular inflammatory reaction was detected. Biodegradation and bioresorption of all implanted HA cylinders was minimal. All implanted cylinders were very firmly attached to the adjacent bony tissue and well incorporated and exhibited excellent osteointegration and osteoconductivity.

**Figure 5:**
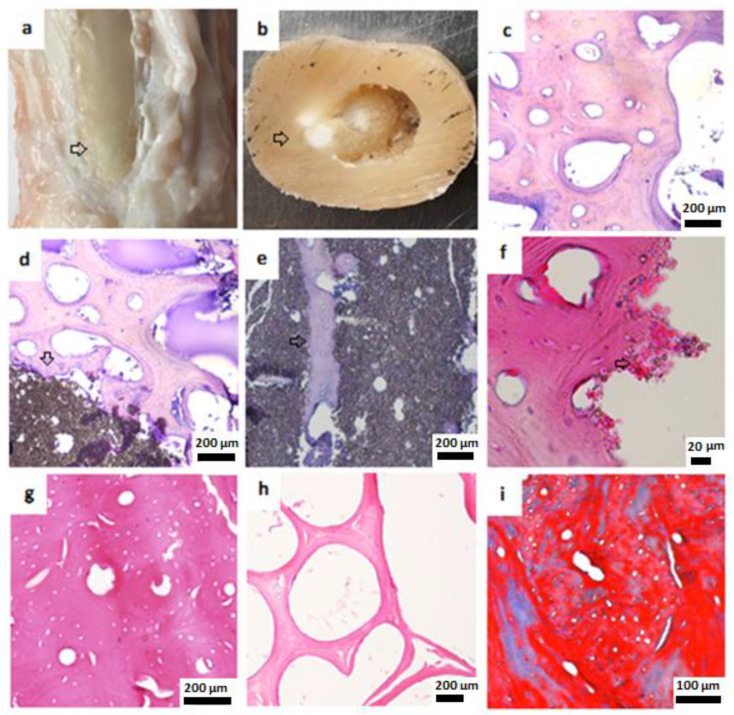
Macroscopic and histological examination of bone tissue 6 months after HA cylinder implantation. (**a**) Macroscopic view, metarsal bone after removal of soft tissue, arrow: the place where new bone was formed. (**b**) Macroscopic view, cross section of the metatarsal bone, arrow: unresorbed parts of HA cylinder. (**c**) Adjacent healthy cortical bone, haematoxylin–eosin staining (H&E), 200 µm. (**d**) Cortical bone, H&E, arrow: HA cylinder, 200 µm. (**e**) Islets of bone tissue in HA cylinder, H&E, 200 µm, arrow shows new bone formation inside the HA cylinder. (**f**) New bone formation around HA cylinder, H&E, 20 µm. (**g**) Newly formed cortical bone, H&E, 200 µm. (**h**) Newly formed trabecular bone, H&E, 200 µm. (**i**) Newly formed cortical bone, Masson’s trichrome, 100 µm.

**Figure 6:**
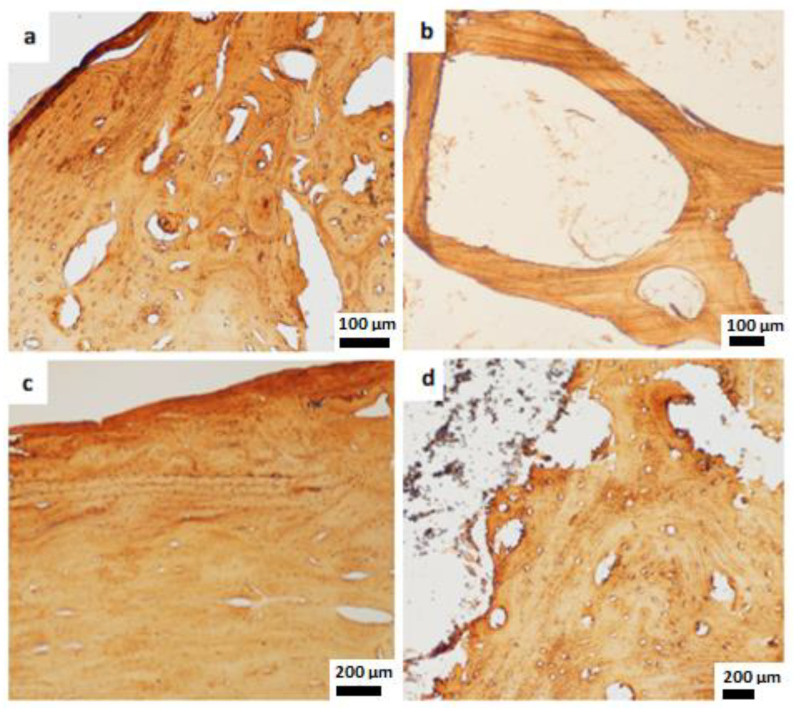
Immunohistochemical staining. (**a**) COL1 expressed in the newly formed cortical bone, 100 µm. (**b**) COL1 expressed in the neoformed trabecular bone after HA cylinder implantation, 100 µm. (**c**,**d**) The positive COL1 staining in newly formed cortical bone after HA plates implantation, 200 µm.

At the surface of HA cylinders, a new bone in close connection with the adjacent bone was seen. The newly formed bone was observed particularly at the periphery of the HA cylinders, and toward their centres, a number of bony tissue islets with osteocytes were seen, giving potential osteoinductive properties to the biomaterial (Figure 5). In all cases, complete bony bridging of the defect with a sporadically uneven surface of the newly formed bone was observed. The newly formed bone tissue exhibited typical cortical and trabecular organization. In some places, we observed the presence of an immature bone. Numerous osteons indicated high remodelling processes in the new bone. A positive immunohistochemical staining for COL1 showed that COL1 was strongly expressed in the cortical bone matrix as well as in the trabecular bone (Figure 6a,b).

#### 3.3.2. HA Plates

Based on our macroscopic and histological evaluation, no signs of inflammatory response were observed; therefore, the HA plates may be considered biocompatible.

**Figure 7:**
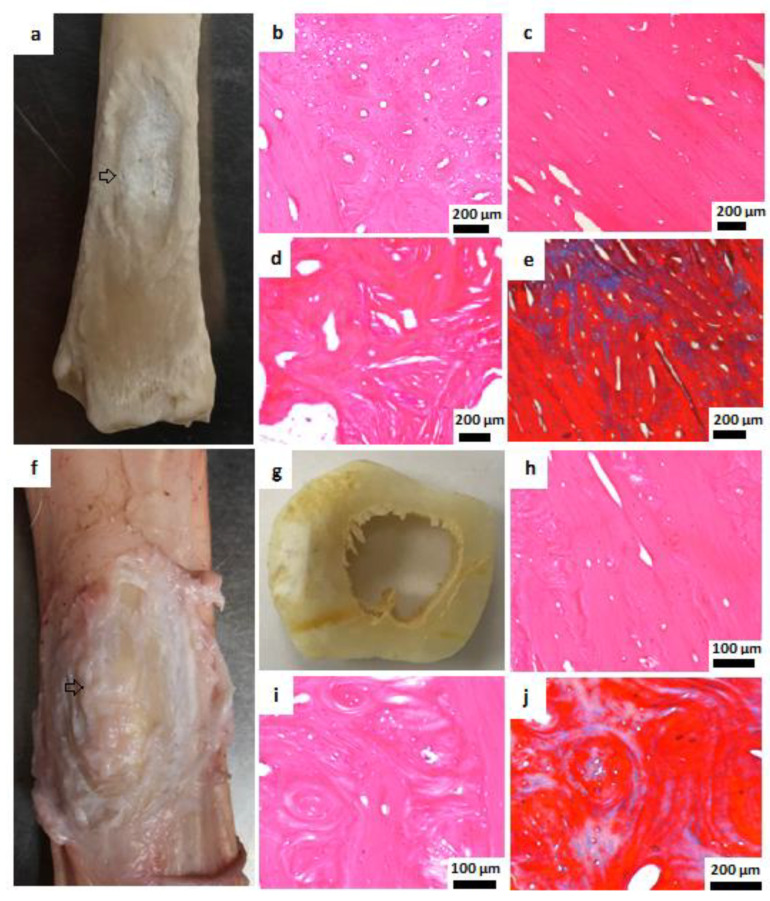
Macroscopic and histological examination of bone tissue 6 months after HA plates implantation. (**a**) Macroscopic view, metatarsal bone, arrow: complete resorption of perforated HA plate and new bone formation. (**b**–**d**) Newly formed cortical bone, H&E, 200 µm. (**e**) Masson’s trichrome staining, 200 µm. (**f**,**g**) Macroscopic view, unresorbed nonperforated HA plate covered by new bone tissue. The arrow points to the defect site in which new bone has formed over the nonperforated plate (**h**,**i**) New cortical bone, H&E, 100 µm. (**j**) New cortical bone, Masson’s trichrome staining, 200 µm.

Complete resorption of the perforated HA plate was visible in two cases (Figure 7). No remnants of the biomaterial used were observed and the complete formation of new bone was visible. At the defect margins, excellent bonding of the new bone to the adjacent bone was observed. However, the surface of the newly formed bone tissue was slightly uneven, and the margin of the newly formed tissue slightly protruded from the surface. In one case, a small amount of the perforated nonresorbed plate was visible. No visible biodegradation of the nonperforated plates was observed. A thin bridge of the cortical bone covered the nondegraded and nonresorbed HA plates (Figure 7). In all cases, the implants were totally integrated into the surrounding cortical bone without interposition of fibrous tissue. The plate–host bone interface indicated excellent integration of the plates. Macro- and microscopic observations did not reveal presence of fibrous capsules around the plates. The process of bone remodelling was still going on at a high rate, as was demonstrated by the presence of large secondary osteons in various stages of formation. The immunohistochemical analysis showed the presence of COL1 in newly formed bony tissues of all samples. The COL1 was expressed in the bone matrix of the cortical bone. More noticeable distribution of COL1 was observed at the edges of the newly formed tissue (Figure 6c,d).

### 3.4. Ca/P Ratio

The Ca/P ratio obtained using SEM-EDX analysis in newly formed bone was 1.56 ± 0.05 (mean ± SD) after application of the HA plates, 1.58 ± 0.04 after application of the HA cylinders, and 1.55 ± 0.05 (mean ± SD) was in native bone. The resulting comparison suggests that they are not statistically different (*p* > 0.82).

### 3.5. Radiographic Analysis

**Figure 8:**
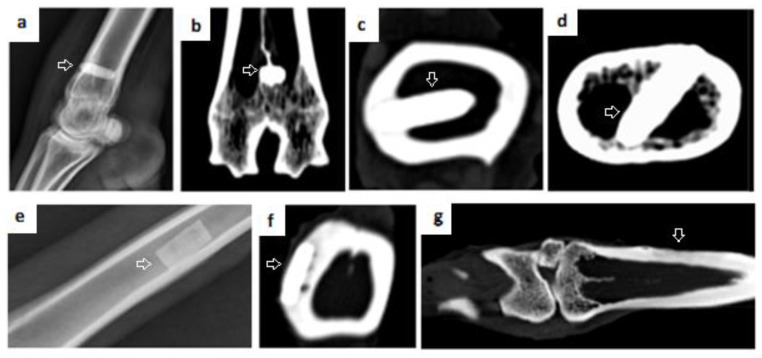
X-ray and CT scans 6 months after implantation. (**a**) X-ray of metatarsal bone after HA cylinder implantation (**b**–**d**) CT scans after HA cylinder implantation. (**e**) X-ray after application of nonperforated plate. (**f**) CT scan of nonperforated HA plate fixed in metatarsal bone. Arrows in figures (**a**–**f**) point to the site of the defect and the implant that was not resorbed. (**g**) CT scan of new bone formed after resorption of the perforated HA plate, arrow: shows newly formed bone tissue after HA plate resorption.

Radiographic analysis was performed to support the broad characterization of the healing process. The correct position of the implants was verified in all animal groups with X-ray images immediately after surgery. The X-ray analysis performed 6 months later revealed no shift of the implants and demonstrated a stable bone bond between the HA ceramics and the host bones. The interface of the HA cylinders and HA plates and the surrounding cortical and trabecular bone tissue were free of inflammatory changes and no irritation of the surrounding tissue was noted. In accordance with the histological results, CT analysis indicated new bone formation in the defect area and around the nonresorbed biomaterials. In all samples, complete cortical bone bridging of the defect site was observed. The bone tissue reached the surface of the implants, with no apparent gap between implants and the new bone tissue. Neither fibrous encapsulation nor focal osteolytic and osteosclerotic changes were detected in the surrounding tissue (Figure 8).

**Figure 9:**
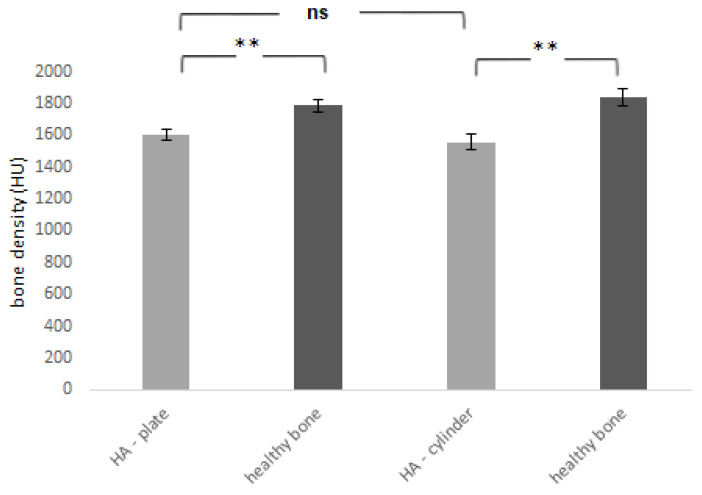
Bone density of newly formed bone tissue 6 months after the application of HA plate and HA cylinder was compared with the density of the adjacent healthy bone. HU—Hounsfield units. ** = *p* < 0.01, ns—nonsignificant = *p* > 0.05.

The average density of the newly formed cortical bone in comparison with the adjacent physiological bone is shown in Figure 9. In all cases, mineralization of the newly formed cortical bone did not reach the stage of complete mineralization of the native adjacent bone (Table 1). Compared to the native healthy bone, the density of the newly formed bone was significantly lower (*p* < 0.01). The fact that the newly formed bone still has a lower density compared to the surrounding healthy bone is physiological. Bone mineralization is gradual, the amount of Ca^2+^ in the bone increases and the bone density measurable on CT also gradually increases.

## 4. Discussion

HA is one of the widely and regularly used bioceramics for bone regeneration, which has been investigated due to its excellent biological properties, potential resorbability, moulding capabilities, and easy manipulation. The use of various types of HA was described in diverse scientific sources [37,42]. HA is characterised by excellent biocompatibility with bony tissue, high bioactivity, and osteoconductivity. HA biomaterials have shown an increased effect on the osteogenic differentiation and proliferation rate of mesenchymal stem cells and a positive influence on osteogenesis [42,43].

Ansari et al. [8] reported that HA, besides other calcium phosphates, is frequently used for its biocompatibility and the potential to simulate the natural mineral portion of bones. Many other studies indicated an excellent biocompatibility of HA with bony tissue [10,35,38,42,43,44]. The biocompatibility of HA obtained from fish waste was also confirmed in vivo and in vitro in a study by Prado et al. [37]. Giorno et al. [38] stated that HA is a biomaterial whose rejection is only minimal or nonexistent. However, the results of their study showed a mild granulomatous and inflammatory response with the development of a fibrotic and collagenous capsule after implantation of HA-based cylinders containing 1% zinc and lead ions. HA ceramics used in our study can be considered highly biocompatible and without ability to cause an inflammatory reaction. We did not observe signs of pathomorphological changes. Histomorphological analysis demonstrated no adverse tissue or inflammatory response. No fibrous capsule and no cellular inflammatory responses were detected at the bone–implant interface.

Many various factors are involved in the gradual biodegradation and bioresorption of bioceramics, including physicochemical solution-mediated processes and the effects of multiple cells [10,45]. Some of these cells degrade bioceramics by phagocytotic mechanisms (monocytes/macrophages, osteoblasts) or by an acidic mechanism that lowers the pH of the environment and enables resorption of the present substrate (osteoclasts). Various mesenchymal cells, such as fibroblasts, are also involved in the process of bioceramic degradation and can induce the solubilisation of ceramics [46]. The cellular mechanism of the ceramic biodegradation process is modulated by several parameters, e.g., the intrinsic properties of bioceramics (particle size, chemical composition and preparation conditions, crystallinity, porosity), the area of implantation, as well as the environment at the implantation site (the presence of multiple proteins, hormones, cytokines, vitamins, ions) [33], and other circumstances (age, sex, and general metabolic health) [10]. Numerous cells involved in the complex mechanism of biodegradation can act directly or indirectly through their growth factors and cytokine secretions and their sensitivity to these molecules [46]. While the perforated HA plates used in our study were completely degraded in two cases and incompletely in one case, the nonperforated ones did not undergo biodegradation and bioresorption. Biodegradation and bioresorption of HA cylinders was only minimal. This indicated that different surface structure and shape significantly influenced the biodegradation processes of HA cylinders and HA plates. Brum et al. [35] contemplated that HA may have a lower degradation rate than other kinds of biomaterials. Klein at al. [47] reported that sintered hydroxyapatite materials showed no detectable resorption over a period of 9 months of implantation. In the study by Yang et al. [43], no significant degradation of the Zn–HA composite during the implantation period was reported. A homogeneous and slow degradation progress was observed. The low solubility of HA was also reported in the study by Cao et al. [48]. The authors of this study stated that the HA-based biomaterials are more suitable for long-term surgical and clinical applications. Biodegradation of HA cylinders and TCP cylinders implanted in the proximal tibia and distal femur was investigated by Eggli et al. [49]. The diameter of the implants was 3 mm, and 15 New Zealand White rabbits were used in this 6-month study. While TCP cylinders were degraded by up to 85.4% after six months, HA cylinders showed only 5.4% volume reduction. Further, the authors compared the HA ceramics with a pore size range of 50–100 microns and 200–400 microns. HA cylinders with smaller pores were completely infiltrated by bone after only four months, whereas in the HA cylinders with larger pores, bone tissue did not penetrate all pores and the amount of bony tissue in the implant after six months was small [49].

Porosity is one of the key features of biomaterials design for bone regeneration. Some critical aspects concerning the clinical success of bioceramics, (e.g., rate of resorption, angiogenesis, tissue ingrowth) depend on the intrinsic properties of the biomaterial but also on the shape, amount, and size of the pores [50]. HA has a porous structure which is comparable to the cancellous bone. The remodelling of HA can lead to mature bone formation [51]. The differences in porosity and roughness could impact dissolution and degradation process of the materials used for implantation [52,53]. According to Hing [54], the degradation of a porous surface could lead to a rapid release of Ca^2+^, which is one of the key factors that facilitate angiogenesis. A porous and rougher surface may be more appropriate for adsorption of biologically active molecules (bone morphogenetic proteins, growth factors). These factors support the attachment, differentiation, and proliferation of cells. Biomaterial degradation and subsequent dissolution depend also on the Ca/P ratio. A decrease in this ratio causes an increase in the solubility of HA in water [55]. Higher dissolution of HA results in the release of more Ca^2+^ and P ions, which can positively affect bone regeneration [56]. In the study conducted by Tanaka et al. [57], the unidirectional porous HA scaffold was inserted into mouse calvarial defects to evaluate the bone-forming ability. The unidirectional porous HA is the scaffold with pores continuously connected in the axial direction. This HA-based biomaterial with 84% porosity showed a high cell number and excellent formation of new bone.

Willie et al. [58] reported bone tissue ingrowth of porous implants inserted into the distal femur of sheep and also pointed to the fact that sheep and humans have a similar pattern of bone ingrowth into porous implants over time. Results of the mentioned study confirmed that the ovine model contributed to understanding of the skeletal attachment of porous-coated implants to the cancellous bone in humans. Triply periodic minimal surface (TPMS) hydroxyapatite implants were used in the repair process of rat femoral bone defects in a study by Charbonnier et al. [59]. Implants with gyroid porosity (GP) and implants with gyroid porosity reinforced by a cortical-like outer shell (GPRC) were specifically designed and created. The integrity of the implants as well as their ability to support bone ingrowth were evaluated 4, 6, and 8 weeks after implantation. The results of this study showed improved mechanical resistance of the GPRC implants and altered osteogenic mechanism compared to GP implants. Bone tissue was found around and inside of both implants, but it was preferentially formed in the area adjacent to the defect boundaries of the GP implants and in the implant core for the GPRC implants. No foreign body reaction or inflammation was detected in and around the implants.

HA ceramics used in our study had appropriate surface properties for the bone tissue growth and also showed excellent osteoconductive properties. The surface of the nonperforated and nonresorbed HA plates, as well as the surface of the HA cylinders, was completely covered with new bone tissue in all cases. Islets of bone tissue were observed also inside the HA cylinders, although the biodegradation of the HA cylinders was minimal. We indicated a potential osteoinductive character of the HA cylinders. The study by Chu et al. [44], conducted in rabbits, investigated three types of biomaterials for bone tissue replacement: HA–20vol%Ti, Ti–metal, and a dense HA ceramic. After a 3-month observation, they concluded that the HA–Ti composite exhibited better osseointegration and osteoconduction properties than the Ti–metal and dense HA ceramic, and it was considered a promising biomaterial for hard tissue replacement. The study by Machado et al. [60] evaluated the biocompatibility and osteoconduction in surgical defects filled with nanohydroxyapatite microspheres containing 1% strontium (nano-SrHA) and stoichiometric nano-HA microspheres (nano-HA) compared to the clot (control) in an animal model of sheep. Three perforations with a diameter of 2 mm were made on the medial surface of the tibia. After 30 days, all groups showed new bone formation from the periphery to the centre of the defect, while it was less intense in nano-SrHA group. A discrete mononuclear inflammatory infiltrate was observed in all groups but both materials were considered biocompatible and osteoconductive.

In the study by Zhao et al. [61] composite scaffolds with HA were used for the repair of rabbit tibia bone defects. Results of this study indicated that the scaffolds had excellent osteoinductivity and osteoconductivity and remarkably promoted new bone formation without any adverse effects.

The development of bioceramics with osteoinductive capabilities has recently been a very important achievement in the field of CaP. Various CaPs have shown osteoinductive properties in that they have the potential to initiate differentiation of undifferentiated cells towards the osteogenic lineage. This leads to formation of new bone, even without exogenous bone morphogenetic protein [50]. The mechanism of osteoinduction has not yet been fully elucidated. Several factors are involved in this process, e.g., the chemical composition, macroporosity, size and geometry of pores, microporosity, microstructure, and surface area [50,62,63]. One of the acceptable hypotheses combines the natural ability of CaP to bind bone morphogenetic proteins with the presence of scaffold concavities that promote retention of bone morphogenetic proteins and ions in the scaffold environment, creating a convenient area for the mesenchymal stem cells differentiation [50]. Li et al. [64] reported that HA has the ability to promote the osteogenic differentiation of stem cells and thereby accelerate the regeneration process of bone. This study further points to a rat calvarial repair model to observe the degradation–diffusion–reconstruction behaviour of HA in the bone-repair process. The authors firstly demonstrated the degradation of HA, followed by the diffusion of the degraded product, and finally reconstruction so that new HA was formed to repair the bone defect. In the study by Lin et al. [65], pluripotent mouse stem cells exposed to HA have expressed some osteo-specific genes. This is consistent with the proposition that the interaction of HA–cell occurs through an osteoinductive potential capable of promoting differentiation into osteoblasts. Ressler et al. [66] also stated that HA can enhance osteogenesis and improve bone regeneration processes.

The osteogenic ability of four different ceramic constructs was observed in a study by Viateau et al. [67]. This study compared the osteogenic potential of ceramic scaffolds (Porites coral, Acropora coral, β-TCP and banked bone) with different resorbability. Tissue-engineered constructs were created with or without autologous bone marrow stromal cells and implanted in the ectopic subcutaneous pouch in sheep. New bone tissue formation was higher in the Porites coral and Acropora coral than in other constructs, and a direct correlation between implant resorption and new bone formation was seen. Among the implants, coral scaffolds containing MSCs provided the best results.

In a study of Bensaid et al. [68], a metatarsal bone defect with a clinically relevant volume was reconstructed in an animal model of sheep. A porous coralline-based hydroxyapatite scaffold in combination with mesenchymal stem cells (MSCs) was used for implantation. Results were compared with coral/MSCs scaffolds and autografts. At 4 months, both constructs had the same osteogenic potential as autologous bone grafts in terms of the amount of new bone (*p* = 0.89). The bone defect was completely replaced by newly formed bone within 14 months. The rate of bone healing was improved when coralline-based HA/MSCs scaffolds were used (the defect healed in five out of seven animals) compared with the coral–MSCs construct (one out of seven healed), but it was still less satisfactory than using autografts (five out of five animals healed). A study by Viateau et al. [69] performed in a sheep model demonstrates the successful treatment of segmental long-bone defects using standardised particulate bone constructs engineered from coral granules and in vitro expanded autologous MSCs. The results were compared with the implantation of an autologous bone graft and a coral scaffold alone. The authors found nonsignificant differences between the amount of new bone in defects filled with coral granules/MSCs bone construct and bone defects filled with autograft, with radiological scores significantly different between the two groups (21% and 100%). The osteogenic ability of the coral/MSCs bone construct is similar to bone autografts. The authors also pointed out that the bone construct preparation procedure is much simpler compared to the preparation of customised massive constructs using computer-assisted techniques. Two different types of scaffolds were tested in a study by Kon et. al. [70], where one of them was made of ion-doped HA/β-TCP and the other was made of undoped HA only. These HA-based scaffolds with a hierarchically organised structure developed through a biomorphic transformation process and showed good results after application in a segmental bone defect in a metatarsal shaft of sheep in terms of safety, osteoinductivity, osteoconductivity, osteointegration, vascularization, and mechanical performance. Histological evaluation did not show any fibrous encapsulation or inflammatory processes at the bone–scaffold interface in both groups. Both scaffolds were well-integrated with the adjacent host bone. The ion-doped HA/β-TCP group showed earlier new bone formation, visible after 3 months. Implant resorption at CT and radiography appeared as gradual fragmentation with no significant differences between the two groups.

The capacity of HA macroporous ceramic cylinders to support large defect repair in the tibia of sheep was investigated by Marcacci et al. [71]. Adjacent bone tissues showed adequate integration of ceramic with newly formed bone as early as 20 days after surgery. After 4 months, extensive integration of the HA ceramic with bone tissue, as well as the formation of compact appearing bone, was detected radiographically and confirmed by morphological study. Sufficient bone growth occurred to allow recovery and more than 80% of the surface of the HA cylinder was covered with new bone tissue. Transverse sections taken through the ceramic cylinders revealed bone formation inside the central canal of the cylinder. A regular lamellar organization of Haversian systems was found in the new compact bone and in most of the bony trabeculae. Areas of woven bone, especially limited to the bone–ceramic interface, represented a smaller part of bone, which is also consistent with our study. The data from this study showed that large defects in a weight-bearing long bone can be repaired with full functional restoration.

## 5. Conclusions

In conclusion, we can state that the HA cylinders and HA plates used in our study, prepared using a tape-casting method in 3D-printed forms, had a positive impact on new bone formation. Both biomaterials were highly biocompatible, osteoconductive, and were well osteointegrated, with potential osteoinductive properties. The ability of biodegradation and bioresorption of these biomaterials is substantial. All HA plates and HA cylinders were not broken or damaged, so their mechanical properties are also of great importance. We consider HA cylinders and HA plates to be suitable biomaterials for further research and potential use in human medicine.

## Figures and Tables

**Table 1:** Bone density.

	a	a’	b	b’
Bone density (HU) mean ± SD	1607 ± 81	1789 ± 96	1559 ± 127	1839 ± 129

(a) Density of the newly formed bone after HA plate application, (a’) density of adjacent healthy bone, (b) density of newly formed bone after HA cylinder application, (b’) density of adjacent healthy bone.

## Data Availability

Data are contained within the article.
